# Investigating the effects of climate variations on bacillary dysentery incidence in northeast China using ridge regression and hierarchical cluster analysis

**DOI:** 10.1186/1471-2334-8-130

**Published:** 2008-09-25

**Authors:** Desheng Huang, Peng Guan, Junqiao Guo, Ping Wang, Baosen Zhou

**Affiliations:** 1 Department of Mathematics, College of Basic Medical Sciences, China Medical University, Shenyang 110001, PR China; 2 Department of Epidemiology, School of Public Health, China Medical University, Shenyang 110001, PR China; 3 Liaoning Provincial Center for Disease Control and Prevention, Shenyang 110005, PR China; 4 Division of Infectious Diseases Control, Shenyang Municipal Center for Disease Control and Prevention, Shenyang 110031, PR China

## Abstract

**Background:**

The effects of climate variations on bacillary dysentery incidence have gained more recent concern. However, the multi-collinearity among meteorological factors affects the accuracy of correlation with bacillary dysentery incidence.

**Methods:**

As a remedy, a modified method to combine ridge regression and hierarchical cluster analysis was proposed for investigating the effects of climate variations on bacillary dysentery incidence in northeast China.

**Results:**

All weather indicators, temperatures, precipitation, evaporation and relative humidity have shown positive correlation with the monthly incidence of bacillary dysentery, while air pressure had a negative correlation with the incidence. Ridge regression and hierarchical cluster analysis showed that during 1987–1996, relative humidity, temperatures and air pressure affected the transmission of the bacillary dysentery. During this period, all meteorological factors were divided into three categories. Relative humidity and precipitation belonged to one class, temperature indexes and evaporation belonged to another class, and air pressure was the third class.

**Conclusion:**

Meteorological factors have affected the transmission of bacillary dysentery in northeast China. Bacillary dysentery prevention and control would benefit from by giving more consideration to local climate variations.

## Background

Shigella infections remain an important public health problem in China, especially among children and old people [1,2]. Bacillary dysentery is an infectious disease of the intestinal tract caused by bacteria of the genus Shigella and is spread by contact with patients or carriers and though food or water contaminated by their feces. The increases in temperature have been observed in most countries and China is no exception[3]. The effects of climate variations on bacillary dysentery incidence have gained more concerns recently. Several studies have explored the association between diarrhoeal diseases and climate variation [4,5]. For example, temperature, rainfall and relative humidity directly affect the rate of replication of bacterial and protozoan pathogens, they may also have an impact on the environmental reservoirs. Many existing studies neglect the multi-collinearity among meteorological factors which affected the accuracy of correlation with bacillary dysentery incidence.

As a remedy, the improved method, ridge regression was suggested and applied. It uses a revised Least Square method to handle correlated predictors by allowing a small amount of bias in the estimates of the coefficients. And due to the fact that many climate factors may be unnecessary for predication, hierarchical cluster analysis was adopted to decompose correlations into different pieces for grouping variables. Based on the combination of these above two methods and existing surveillance data (1950–1996), the present study aims to investigate the effects of climate variations on bacillary dysentery incidence in northeast China.

## Methods

### Study area and data collection

**Figure 1:**
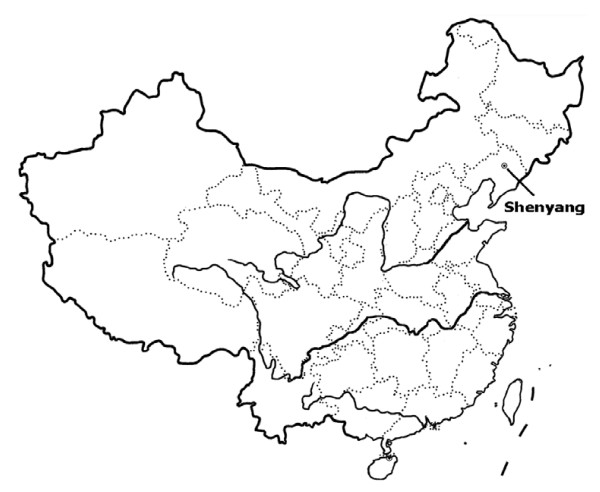
Location of study area (Shenyang) in China.

Shenyang, capital city of Liaoning Province and also the largest city in northeast China, 41°N and 123°E (Figure 1), was selected as the study area. It has a temperate climate and a population of about 7.6 million. Shenyang has a semiwetness continent climate of the north temperature zone as well as concentrated precipitation and distinct seasons because of the monsoon. Annual average temperature is about 8.1°C, the highest monthly average temperature is 24.0°C in July, and the lowest monthly average temperature is -8.5°C in January. Its annual rainfall is 501.5 mm and the non-frost period is 183 days. Four seasons are spring, March – May; summer, June – August; autumn, September – November; winter, December – February. Demographic information for Shenyang was collected from local government report.

Bacillary dysentery is a legally mandated notifiable disease in China, the Law on the Prevention and Control of Infectious Diseases [6] requires health-care staff to report any of the 37 infectious diseases, including bacillary dysentery, to the Center for Disease Control and Prevention (CDC) through the National Noticeable Infectious Disease Reporting system (NIDR). The data of monthly incidence of bacillary dysentery in Shenyang from 1950 to 1996 was obtained from Liaoning Center for Disease Control and Prevention. Because the study period covers a long time span, four time subgroups were obtained based on historical and economic development of China. In order to control seasonal effects, two models were set up in each time subgroup, one is based on data from January to July, and the other is base on data from August to December. The meteorological information was collected from Shenyang Meteorological Bureau. Meteorological data consisted of the corresponding monthly air pressure, average temperature, maximum temperature, minimum temperature, precipitation, evaporation and relative humidity.

### Data analysis

#### Correlation analysis

The relationship between monthly mean meteorological factors and the monthly incidence of bacillary dysentery was examined. Spearman's correlation was performed to quantify the relationship between monthly weather variables and the monthly incidence of bacillary dysentery a lag of one to four months.

#### Multi-colliearity Diagnosis

The judgment of multi-colliearity was made by checking related statistics, such as tolerance value or variance inflation factor (VIF), and condition index. The *i*th tolerance value is defined as 1-$R_{k}^{2}$, where $R_{k}^{2}$ is the coefficient of determination for regression of the *i*th independent variable on all the other independent variable. VIF is just the reciprocal of a tolerance value. With the recommendations of several statisticians[7], large VIF value greater than 10 and/or average VIF greater than 6 indicates strong collinearity, and that those variables are collinear if their condition indexes are more than 20 and corresponding variance proportions are more than 0.5.

#### Ridge regression analysis

There was collinearity among the meteorological factors, especially between constant and air pressure, average temperature and maximum temperature, and minimum temperature and relative humidity. To avoid the multi-collinearity, ridge regression was used to quantify the relationship between weather variables and bacillary dysentery incidence. By using an improved least square method, ridge regression sought standardized coefficients ${\tilde{b}}_{\text{i}}$ (i = 1, 2,..., m), its formal equation was written as:

(1) $$\{\begin{array}{c} (1+k){\tilde{b}}_{1}+r_{12}{\tilde{b}}_{2}+\cdots +r_{1m}{\tilde{b}}_{m}=r_{1(m+1)}\\ r_{21}{\tilde{b}}_{1}+(1+k){\tilde{b}}_{2}+\cdots +r_{2m}{\tilde{b}}_{m}=r_{2(m+1)}\\ \cdots \cdots \cdots \cdots \cdots \cdots \cdots \cdots \cdots \cdots \cdots \cdots \cdots \cdots \cdots \\ r_{m1}{\tilde{b}}_{1}+r_{m2}{\tilde{b}}_{2}+\cdots +(1+k){\tilde{b}}_{m}=r_{m(m+1)} \end{array}$$

Compared to Ordinary Least Square(OLS) linear regression, the basic principle of ridge regression is to artificially reduce correlation coefficient r_ij _of each pair of variables x_i _and x_j _(including response variable and independent variables) to r_ij_/(1+k), k is called ridge parameter, and usually 0 < k < 1. K value was selected when all the regression coefficients were relatively stable and the sign of the coefficients did not change.

#### Hierarchical cluster analysis

Hierarchical cluster analysis was adopted to group the weather variables. In this study, the agglomerative method with between-group average linkage algorithm was adopted and the measure for similarity was the Pearson correlation.

All the above statistical analyses were performed by Statistical Product and Service Solutions (SPSS 12.0 for windows, SPSS Inc., Chicago, IL, USA).

#### Ethical review

The present study was reviewed by research institutional review board of China Medical University and found to be utilization of disease surveillance data and meteorological data not requiring oversight by an ethics committee.

## Results

### Statistical description of bacillary dysentery in Shenyang city

**Figure 2:**
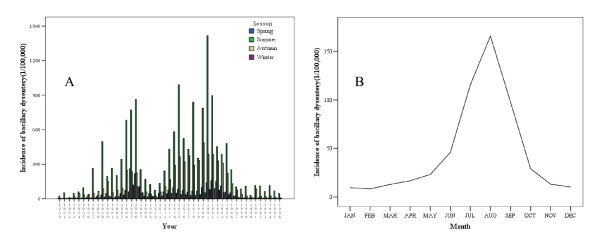
**Distribution of bacillary dysentery incidence (Shenyang, China, 1950–1996). **A) Long-term trend. B) Seasonal distribution.

The distribution of bacillary dysentery incidence in Shenyang between 1950 and 1996 is shown in Figure 2. Figure 2a shows the long-term trend, which is that the incidence of bacillary dysentery in Shenyang rose over periods 1950–1966 and 1970–1981, and declined over periods 1966–1970 and 1981–1996. Figure 2b indicates that there was a seasonal distribution of bacillary dysentery in the study area, with about 85% of cases occurring in summer and autumn The incidence of bacillary dysentery increased from January to August and decreased from August to December.

### Spearman's correlation analysis

The relationship between monthly mean meteorological factors and the monthly incidence of bacillary dysentery in the studied period was examined by Spearman's correlation analysis (Table 1).

**Table 1:** Spearman correlations between monthly incidence of bacillary dysentery and corresponding monthly meteorological factors (1950–1996, Shenyang, China)

Year	Air pressure	Average air temperature	Maximum temperature	Minimum temperature	Precipitation	Evaporation	Relative humidity
1950–1966	-0.459	0.542	0.521	0.554	0.429	0.419	0.194
1967–1976	-0.715	0.772	0.768	0.790	0.657	0.545	0.647(lag1)
1977–1986	-0.697	0.824	0.827	0.825	0.711	0.641	0.604
1987–1996	-0.750(lag1)	0.861	0.846	0.870	0.791	0.602	0.601(lag1)
1950–1996	-0.493	0.580	0.568	0.593	0.506	0.374	0.332

All correlation coefficients are significant at the 0.01 level (2-tailed)

Lag1: one month prior

Due to the fact that the history data covered more than 40 years, four time subgroups were set for analysis based on historical and economic development of China. Table 1 shows that temperatures, precipitation, evaporation and relative humidity were positively related to the monthly incidence of bacillary dysentery, while air pressure was inversely correlated with the incidence. Lagged effects existed in air pressure from 1987 to 1996 and in relative humidity from 1967 to 1976 and from 1987 to 1996.

### Multi-collinearity Diagnosis

The diagnosis of multi-collinearity is shown in Table 2 and Table 3. In Table 2, the largest VIF is 164.187, and the average VIF is 54.574, therefore, climatic variables are strongly collinear. In addition, Table 3 shows that the largest condition index is 815.54 which also indicates strong multi-collinearity. With the help of condition index and variance proportion, we found that collinearity existed between the constant and air pressure, the average temperature and maximum temperature, as well as minimum temperature and relative humidity.

**Table 2:** Collinearity Statistics for explanatory variables

Variables	Tolerance	VIF
Air pressure	0.235	4.249
Average temperature	0.006	164.187
Maximum temperature	0.009	113.969
Minimum temperature	0.011	87.756
Precipitation	0.388	2.580
Evaporation	0.154	6.505
Relative humidity	0.360	2.775

**Table 3:** Collinearity Diagnostics between explanatory variables

Condition Index	Variance Proportions							
	Constant	Air pressure	Average temperature	Maximum temperature	Minimum temperature	Precipitation	Evaporation	Relative humidity
1.00	0.00	0.00	0.00	0.00	0.00	0.00	0.00	0.00
2.00	0.00	0.00	0.00	0.00	0.00	0.00	0.00	0.00
4.39	0.00	0.00	0.00	0.00	0.00	0.55	0.02	0.00
8.98	0.00	0.00	0.00	0.00	0.01	0.31	0.36	0.02
30.08	0.00	0.00	0.09	0.12	0.39	0.09	0.45	0.43
35.89	0.00	0.00	0.08	0.04	0.56	0.00	0.11	0.54
50.00	0.00	0.00	0.82	0.83	0.04	0.03	0.00	0.00
815.54	1.00	1.00	0.01	0.01	0.00	0.01	0.05	0.01

### Ridge regression analysis

**Figure 3:**
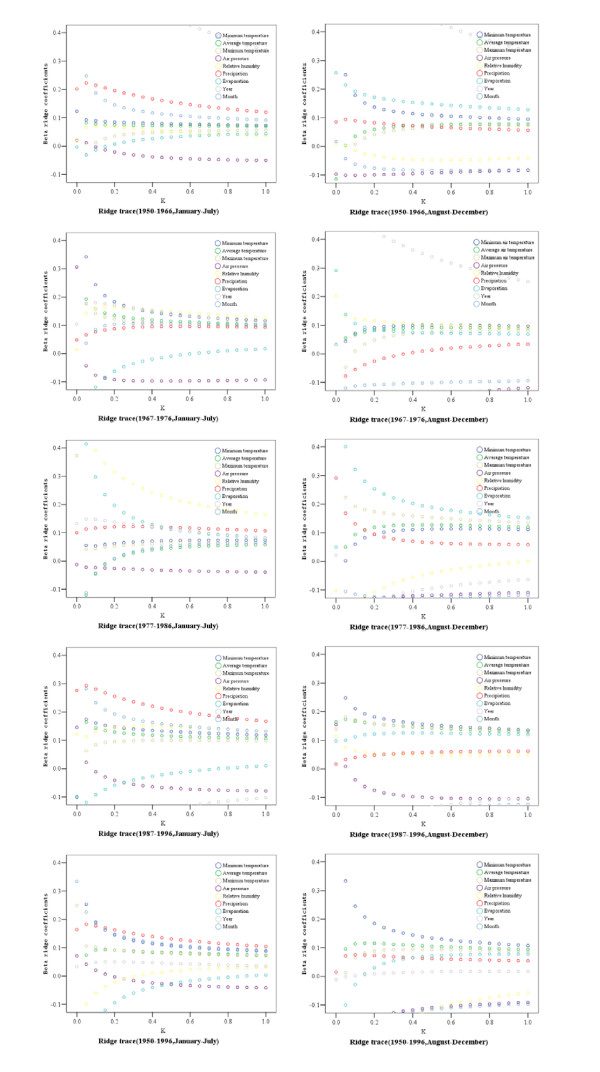
Ridge trace between climatic variables and logarithmic incidence of bacillary dysentery (1950–1996, Shenyang, China).

Ridge regression was adopted to examine the relationship between the monthly mean weather variables and the monthly incidence of bacillary dysentery. Ridge trace maps (Figure 3) show that when ridge parameter *k *reached 0.5, ridge regression coefficients were stable. When ridge parameter was set as 0.5, the ridge regression coefficients were then obtained (Table 4). Different patterns for the analysis unit Sub-grouping improved the determinant coefficients of all models. However, determinant coefficients of each unit differ from 0.490 to 0.892. Most models give the following similar results: relative humidity and precipitation have a positive association with incidence from January to July, and have no association from August to December, while evaporation is the opposite. Some results vary different for the four time subgroups, for example, ridge regression coefficients are not always statistically significant. And also, lagged effects only existed in air pressure from 1987 to 1996 and in relative humidity from 1967 to 1976 and from 1987 to 1996. In order to compare variables effects between models, original variables without lagged values were used for ridge regression analysis directly.

**Table 4:** Ridge regression coefficients between monthly climatic variables and logarithmic incidence of bacillary dysentery (with k = 0.5, 1950–1996, Shenyang, China)

Duration	R^2^	Minimum temperature	Average temperature	Maximum temperature	Air pressure	Relative humidity	Precipitation	Evaporation	Long-term Effect	Season
1950–1966, Jan – Jul	0.718	0.079*	0.072*	0.051*	-0.042	0.053	0.156*	0.032	0.458*	0.110*
1950–1966, Aug – Dec	0.818	0.109*	0.075*	0.067*	-0.093*	-0.047	0.068	0.148*	0.442*	-0.084*
1967–1976, Jan – Jul	0.653	0.138*	0.115*	0.106*	-0.097*	0.149*	0.096	-0.009	0.142*	0.106*
1967–1976, Aug – Dec	0.768	0.101*	0.092*	0.076*	-0.156*	0.103	0.013	0.073*	0.338*	-0.101*
1977–1986, Jan – Jul	0.490	0.072*	0.046	0.063*	-0.033	0.222*	0.119	0.114*	0.111	0.052
1977–1986, Aug – Dec	0.822	0.113*	0.127*	0.153*	-0.118*	-0.041	0.065	0.189*	-0.094*	-0.125*
1987–1996, Jan – Jul	0.822	0.131*	0.115*	0.100*	-0.069*	0.148*	0.207*	-0.017	-0.140*	-0.154*
1987–1996, Aug – Dec	0.892	0.154*	0.144*	0.142*	-0.101*	0.048	0.058	0.125*	-0.132*	-0.135*
1950–1996, Jan – Jul	0.306	0.107*	0.082*	0.080*	-0.030	0.018	0.131*	-0.027	0.045	0.111*
1950–1996, Aug – Dec	0.430	0.134*	0.105*	0.094*	-0.110*	-0.116*	0.062	0.071*	0.016	-0.116*

* *P *< 0.05

### Cluster analysis

**Figure 4:**
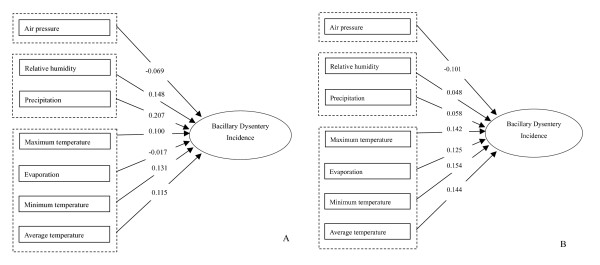
**Combination of cluster analysis and ridge regression analysis between climatic variables and logarithmic incidence of bacillary dysentery (1987–1996, Shenyang, China).** A) 1987–1996, January – July. B) 1987–1996, August – December.

Figure 4 shows the effects of climate variations on bacillary dysentery incidence, the regression coefficients were listed above the arrows. Due to the long period, only the model for 1987 to 1996 was listed. During this period, all meteorological factors were divided into three categories. Relative humidity and precipitation belonged to one class, temperature indexes and evaporation belonged to another class, and air pressure was the third class (Figure 4).

## Discussion

Although incidence of intestinal infectious diseases has declined considerably in developed countries, they are still major public health problems in developing countries, especially in the economic developing regions[8,9]. The transmission of Shigella is determined by many factors, such as human dietary lifestyle, and its strains' susceptibility to drugs and climate variations. Climate variability that impacts the incubation rate of Shigella is considered one of the major environmental contributors to Shigella transmission.

Climate change, especially global warming, has already brought and will continue to bring about challenges to public health and communicable disease control and prevention. The effects of climate variations on bacillary dysentery incidence have gained more recent concern. The historical climatic data from 1950 to 1996 demonstrate a significant increase in temperatures in Shenyang city. It has been reported that temperature was the key climatic indicator in the transmission of bacillary dysentery, a potential 1°C increase in maximum or minimum temperature was associated with up to 12% increase in bacillary dysentery cases in the northern city if other affecting factors remain unchanged[10]. Public health interventions should be developed at this stage to adapt and mitigate the possible consequences of climate change in the future with consideration of local climatic conditions[11]. The model constructed in the present study showed that explanatory climatic variables for the incidence of bacillary dysentery could be divided into three categories, namely relative humidity and precipitation; temperature indexes and evaporation; and air pressure. Among them, only air pressure showed negative correlations, all other variables showed positive correlations. From the point of effect size, temperature indexes and precipitation had greater effect which is of part concordance with the results of other studies in China[12,13]. Precipitation, relative humidity and air pressure may affect the transmission of food-borne diseases by influencing the pathogens and then affect the incidence of bacillary dysentery. The impact of precipitation on diarrhoeal disease is far from conclusion. Different studies obtained inconsistent results [14-16]. The three categories of climatic variables could be used as predictors for the number of bacillary dysentery cases in Shenyang. The results of the present study may be used to assist public health decision making and community health education. Further studies about this issue are necessary.

Due to the multi-collinearity in independent variables, the regression coefficients are very unstable with excessive standard error. A general approach to treat the existence of variables that are non significant to the effect of response variables is to remove them one by one until all the variables reserved in the equation are significant. In the present study, high multi-collinearity existed between the constant and air pressure, average temperature and maximum temperature, minimum temperature and relative humidity. We proposed a modified method to combine ridge regression and cluster analysis to solve this problem. The adoption of ridge estimated method to construct the regression model improved the effect of multi-collinearity, reduced the standard error of regression coefficients and produced more stable results. The stable regression coefficients then made the estimation of effect more reliable.

Though the meteorological factors are nonmodifiable, we can modify our dietary lifestyle, such as reducing intake of raw vegetables or cleaning vegetables and fruits more thoroughly. Local weather forecasting may have policy implications for bacterial dysentery prevention and control. Strengthening the education of public health will help to eliminate or reduce the epidemic of bacterial dysentery.

One limitation of this study was that we only focused on the relationship between the meteorological factors and the incidence of bacillary dysentery. The socio-economic status, especially the awareness of health care has been improved in China. So in order to remove the effect of preventive measures, this study used only the historical data before 1996 and four time subgroups that were obtained based on historical and economic development of China. The changes in social and economic conditions need to be addressed in further studies. Bacillary dysentery is a legally mandated notifiable disease in China, as a provincial capital city, Liaoning provincial CDC and Shenyang municipal CDC have kept good records for Shenyang bacillary dysentery incidence. However, there is still another limitation of the present study, which is that under-reporting is possible in the disease surveillance system, especially before the computer and web were widely applied. An investigation of missing reports of notifiable diseases in China in 2005 indicated that there was much to be improved upon in Chinese medical facilities as far as reporting of infectious diseases[17]. So, the correlation between climatic variation and bacillary dysentery may be underestimated. However, there is no evidence to suggest there was any trend in under-reporting during the four time subgroups in Shenyang city. Therefore it is considered that the impact of under-reporting during each time sub group on the results is not significant.

## Conclusion

In summary, relative humidity, temperature indexes and atmospheric pressure affected the transmission of the bacillary dysentery in Shenyang, located in the northeast of China. Climate variations have brought and will continue to bring about challenges to communicable disease control and prevention. Bacillary dysentery prevention and control should be taken by giving more consideration to local climate variations and integrated modelling based on data systems is necessary to disease control and prevention.

## Competing interests

The authors declare that they have no competing interests.

## Authors' contributions

DH conceived the study, attracted funding and drafted the manuscript. DH, PG and BZ managed and analyzed the data. PW managed the bacillary dysentery incidence database which was administered and supervised by JG. All authors contributed to the writing of the final version of this paper.

## Pre-publication history

The pre-publication history for this paper can be accessed here:


